# Keep Your Mouth Shut

**Published:** 1856-01

**Authors:** 


### KEEP YOUR MOUTH SHUT,

ALL you that will keep late hours these cold winter nights in crowded heated rooms, until animal vigor and mental sprightliness are exhausted, and yet must breast the bleak winds of January to get home. I see nothing amiss in the festivities of friends and neighbors and kindred these long winter evenings: better that. than moping at home; nothing amiss in the glad re-unions of the young and cheery-hearted, even though they may be extended once in a while to the wee short hours ayant the twal. I love to see gladness in all, at any hour of the twenty-four; but to do these things safely and long, make it a practice to observe two or three simple and easy precautions.

Before you leave, bundle up well—gloves, cloak, comforter—shut your mouth before you open the street door, and keep it resolutely closed until you have walked briskly for some ten minutes; then, if you keep on walking, or have reached your home, you may talk as much as you please. Not so doing, many a heart once happy and young now lies in the church-yard, that might have been young and happy still. But how? If you keep your mouth closed and walk rapidly, the air can only reach the lungs by the circuit of the nose and head, and becomes warmed before reaching the lungs, thus causing no derangement; but if you converse, large drafts of cold air dash directly in upon the lungs, chilling the whole frame almost instantly. The brisk walking throws the blood to the surface of the body, thus keeping up a vigorous circulation, making a cold impossible if you don't get into a cold bed too quick after you get home. Neglect of these brings sickness and premature death to multitudes every year. See "BRONCHITIS AND KINDRED DISEASES."

